# An updated calculator for determining levodopa-equivalent dose

**DOI:** 10.1186/s42466-021-00157-6

**Published:** 2021-10-25

**Authors:** D. Nyholm, W. H. Jost

**Affiliations:** 1 Department of Neuroscience, Neurology, Uppsala University, Uppsala, Sweden; 2 Parkinson-Klinik Ortenau, Kreuzbergstr. 12, 77709 Wolfach, Germany

**Keywords:** Parkinson’s disease, Calculator, Levodopa, Levodopa/entacapone/carbidopa intestinal gel infusion

## Abstract

Calculation of levodopa-equivalent dose in Parkinson’s disease has become common in research, but is also a useful tool in clinical practice, especially when initiating device-aided treatments (deep brain stimulation, apomorphine and levodopa infusions). The aim with the present calculator is to provide an updated conversion table, including dose calculation of the recently developed levodopa/entacapone/carbidopa intestinal gel infusion. Future versions of the calculator should be made conducive to learning by means of artificial intelligence.

**Dear Editor**,

In advanced Parkinson’s disease (PD), frequent use is made of deep brain stimulation (DBS) and infusion therapy with levodopa or apomorphine. With these therapies, usually only technical problems are considered for discussion, while in fact the details of adapting the patient’s oral medication are far more difficult and present a number of challenges. When starting DBS or infusion therapies, adjusting and reducing the patient’s existing oral medication is something that needs careful consideration and is not completely straightforward. Generally, patients are given a complex combination of various medications such as levodopa plus decarboxylase inhibitor, dopamine agonists, MAO (Monoamine oxidase) B-inhibitors, COMT (catechol-O-methyltransferase)-inhibitors, and amantadine. Levodopa is the only drug whose dose can be decided relatively easily, but even then the dose must be recalibrated frequently. With some drugs, such as dopamine agonists, the medication should be tapered off slowly to avoid withdrawal symptoms. Recommendations for gradually reducing these drugs are currently lacking [1]. In addition to the problem of tapering, the problem of equivalent dosage is considerably more difficult: At present there are only a few studies on single preparations, not all PD therapies, and particularly not for combinations of several medications. The review by Tomlinson provides a useful tool [2], but unfortunately, the existing gaps have not yet been filled in with empirical data.

A method for dose conversion of drugs to establish the equivalent dosage is crucial when switching from oral medication to apomorphine or levodopa infusion, but also for doing DBS. The need for a simple conversion also increases as there are now different suppliers of apomorphine and two different levodopa pumps: LCIG (levodopa/carbidopa intestinal gel) and LECIG (levodopa/entacapone/carbidopa intestinal gel). Usually calibrations are simply taken over from the recommendations of the manufacturers or from routine practice of the individual clinic. A standardized and generally accepted principle of conversion would be highly desirable: one which is based not only on a simple table but is also capable of learning (artificial intelligence). This is the goal we set for ourselves and we have developed a calculator for computing equivalent doses. The calculator is based on an Excel table which makes use of the data from Tomlinson et al. [2], with addition of newer PD therapies like opicapone, safinamide and extended-release levodopa [4]. This tool is in the process of being published online and should be available free of charge for physicians. Legal issues still need to be clarified, because it has to be certificated as a medical device. As a matter of course, the data are necessarily tentative and have no claim to absolute validity, due to the fact that they depend on the available literature published to date. In a subsequent step, the calculator will be made conducive to learning, and actual as well as future data will be carefully collated in order to generate specific data for when patients start apomorphine infusion or levodopa infusion therapies (LCIG or LECIG). However, this will require large amounts of data which can only be accomplished by including information from a wide range of patients. Therefore we will compare the calculated dose with the clinically necessary dose. One initial step in this direction would entail inputs from the ELEGANCE Registry (A Prospective Non-interventional Study on Long-term Effectiveness and Safety of Levodopa-Entacapone-Carbidopa Intestinal Gel (LECIGon®) in Patients with Advanced Parkinson's Disease in Routine Care). In addition, further information should be gleaned from data on the question of how PD medications are best tapered.

Various conversion tables have already been developed and an app has also been published [3]. We have created an easy-to-use application that allows for the calculation of levodopa equivalent doses and flow rates for levodopa intestinal gel infusion therapies. The levodopa equivalent dose is calculated according to conversion factors and proposed parameters, reported elsewhere, by submitting daily dosages of commonly used anti-Parkinson drugs, single or in combination (Fig. 1) [2, 4, 5]. Flow rates can be determined from manually entered values for a levodopa equivalent dose, oral levodopa preparations and/or previous LCIG or LECIG, taking SmPC recommendations for dosage into account. Some of those values are preset according to recent literature but can be adjusted by the user for broad applicability [5–7]. The user can choose between administration over any chosen amount of time in a day or a 24-h continuous treatment, which allows for a variable duration and flow rate over night (Fig. 2). The proposed dosage must then always be fine-tuned for each individual based on treatment response.

**Fig. 1:**
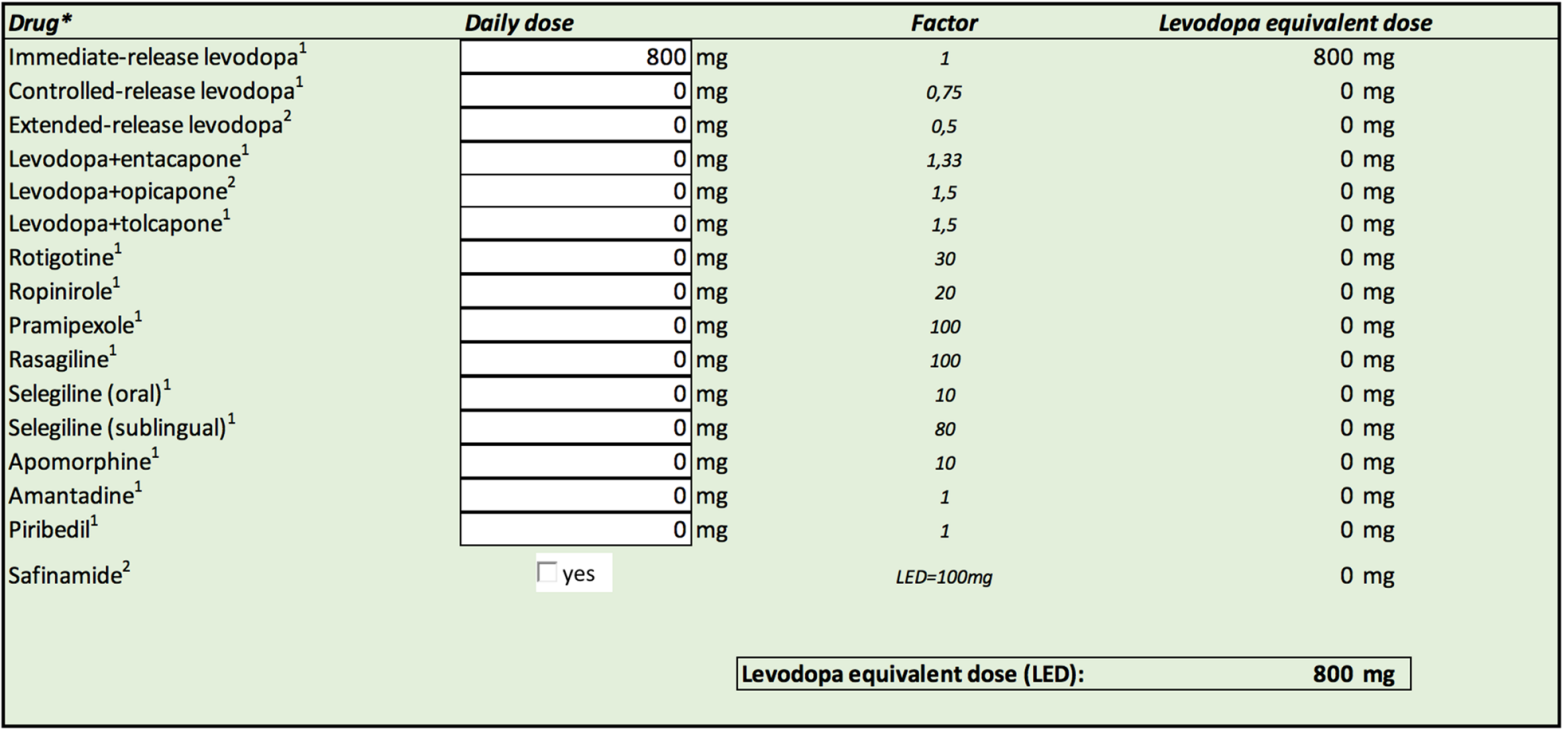
Levodopa equivalent dose is calculated according to conversion factors and parameters reported elsewhere by submitting daily doses of commonly used anti-Parkinson drugs (single or in combination) [2, 4, 5]. Immediate-release levodopa 100 mg 8 times daily is given as an example. Decarboxylase inhibitor in combination with levodopa is understood; difference in bioavailability of levodopa due to presence of benserazide or carbidopa is not considered

**Fig. 2:**
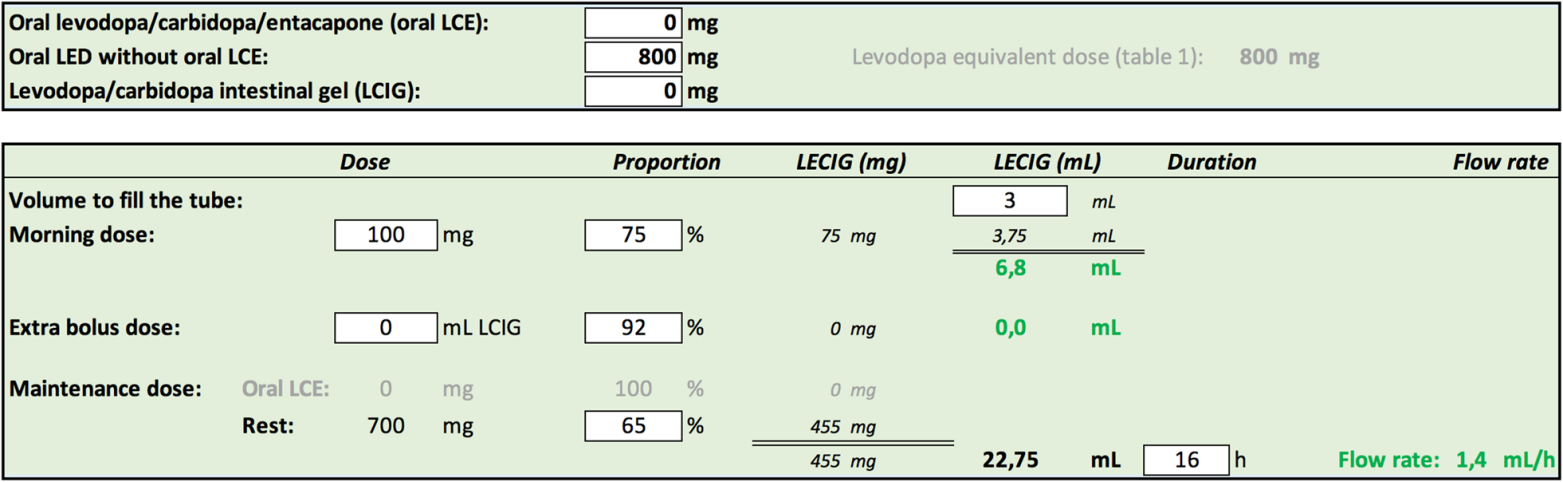
Calculation of LECIG flow rate from the levodopa equivalent dose (LED), oral levodopa/carbidopa/entacapone and/or LCIG. White boxes can be modified by the user. Transition from oral LED of 800 mg is used as an example, resulting in a proposed initiation of LECIG with a morning dose of 6.8 mL and a maintenance dose of 1.4 mL/h. As recommended by the manufacturer, LECIG dose is not reduced after switch from oral levodopa/carbidopa/entacapone and maintenance dose is reduced to 65% after switch from LCIG [5]. Proportional dose reductions are preset according to Senek et al. 2020 [7] and Öthman 2021 [6]

Levodopa is still the most effective drug in PD. Therefore it makes sense to use the levodopa equivalent dose (LED), especially in patients with infusion therapies. The calculator could always be used when performing infusion therapies or when calculating the LED for any other reason.

## Data Availability

Not applicable.

## Abbreviations

PD: Parkinson’s disease
DBS: Deep brain stimulation
MAO: Monoamine oxidase
COMT: Catechol-O-methyltransferase
LCIG: Levodopa/carbidopa intestinal gel
LECIG: Levodopa/entacapone/carbidopa intestinal gel
SmPC: Summary of product characteristics
LED: Levodopa equivalent dose
